# Loss of the epigenetic mark, 5-Hydroxymethylcytosine, correlates with small cell/nevoid subpopulations and assists in microstaging of human melanoma

**DOI:** 10.18632/oncotarget.6062

**Published:** 2015-10-09

**Authors:** Jonathan J. Lee, Martin Cook, Martin C. Mihm, Shuyun Xu, Qian Zhan, Thomas J. Wang, George F. Murphy, Christine G. Lian

**Affiliations:** ^1^ Program in Dermatopathology, Department of Pathology, Brigham and Women's Hospital, Harvard Medical School, Boston, MA, USA; ^2^ Department of Histopathology, Royal Surrey County Hospital, Guildford, United Kingdom; ^3^ Cancer Research UK, Manchester Institute, Manchester, United Kingdom; ^4^ Department of Dermatology, Brigham and Women's Hospital, Harvard Medical School, Boston, MA, USA

**Keywords:** 5-hydroxymethylcytosine (5-hmC), melanoma, maturation, small cell/nevoid subpopulation, pre-existing nevus

## Abstract

Melanomas in the vertical growth phase (VGP) not infrequently demonstrate cellular heterogeneity. One commonly encountered subpopulation displays small cell/nevoid morphology. Although its significance remains unknown, such subpopulations may pose diagnostic issues when faced with differentiating such changes from associated nevus or mistaking such regions for nevic maturation (pseudomaturation). That ‘loss’ of the epigenetic biomarker, 5-hydroxymethylcytosine (5-hmC), is a hallmark for melanoma and correlates with virulence prompted us to explore the diagnostic utility and biological implications of 5-hmC immunohistochemistry (IHC) in melanomas with small cell/nevoid subpopulations. Fifty-two cases were included in this study, including melanomas with small cell/nevoid subpopulations (MSCN) or melanomas with pre-existing nevus (MPEN). Semiquantitative and computer-validated immunohistochemical analyses revealed invariable, uniform loss of 5-hmC in the conventional melanoma component. By contrast, the nevic components in MPEN cases demonstrated strong nuclear immunopositivity. In MSCN cases, there was partial to complete loss of 5-hmC restricted to these nevoid areas. Based on recent data supporting tight correlation between 5-hmC loss and malignancy, our findings indicate a potential ‘intermediate’ biological nature for small cell/nevoid subpopulations. Because 5-hmC assisted in differentiating such regions from associated nevus, the use of 5-hmC as an adjunct to microstaging in difficult cases showing VGP heterogeneity should be further explored.

## INTRODUCTION

Melanoma is an aggressive, malignant neoplasm that arises from the transformation of the neural crest-derived melanocyte. Despite a common cell of origin, melanoma represents a biologically diverse group of malignant neoplasms that vary at the clinical, cytologic, immunophenotypic, genetic, and, potentially, epigenetic levels. Histologically, variants of melanoma have been described, highlighting unique morphologic features that can distinguish between discrete clinicopathologic variants of melanoma [1]. In addition to these inter-tumoral variations, melanoma is also recognized for frequently demonstrating significant cytomorphologic heterogeneity within a single lesion. The most extreme example of this *intra*-tumoral or intralesional heterogeneity is exemplified by melanomas arising in association with a nevus, an event documented in up to 30% of primary cutaneous melanomas and recently shown to have no prognostic impact [2, 3]. Beyond dichotomous expression of nevic precursors juxtaposed with their malignant counterparts, the term ‘polyclonism’ refers to the presence of morphologically distinct subpopulations within the invasive component of a single tumorigenic melanoma [4]. One specific example is when melanoma cells become smaller with increasing dermal depth in a form of nevoid mimicry. This cytomorphologic transition from larger to smaller melanoma cells has been referred to as ‘pseudomaturation’, ‘apparent maturation’, as well as ‘pseudonaevoid phenomenon’ [1, 5, 6]. This phenomenon may occur in up to 8% of cases also has been referred to as ‘paradoxical maturation’ and has also been proposed to reflect a more ‘quiescent’ state, based on the significant reduction of Ki-67 staining within the smaller ‘maturing’ cells [7].

The clinical, pathobiological, and prognostic significance of small cell polyclonisim in melanoma remains incompletely understood. However, one area of practical, diagnostic interest is in the potential effect of this phenomenon on the determination of the prognostically-relevant Breslow depth. When smaller, more nevoid melanoma cells exist at the deepest, invasive front of the lesion, there may be diagnostic confusion as to whether these smaller cells represent benign pre-existing nevus cells or a small cell/nevoid melanoma subpopulation. Accordingly, how such lesions are microstaged may vary based on this interpretation, resulting in inconsistencies in establishing critical prognostic parameters and related clinical management.

In previous studies, we have shown that the functionally-active epigenetic biomarker, 5-hydroxymethylcytosine (5-hmC) as detected through immunohistochemistry (IHC), is strongly expressed in normal melanocytes, is partially lost in melanocytic dysplasia, and is completely absent in melanoma [8, 9]. Loss of 5-hmC reflects a deficiency in the Ten-Eleven Translocase (TET) family of active DNA de-methylation enzyme activity [10, 11]. Restoration of the 5-hmC landscape *via* TET overexpression has been shown to inhibit melanomagenesis in experimental xenograft models, suggesting a potential tumor suppressive role of TET2 function and 5-hmC [8]. In addition, IHC for 5-hmC has shown diagnostic, biomarker value with a sensitivity and specificity of up to 93% and 98%, respectively, in differentiating between benign cutaneous nevi *versus* melanoma and potentially higher values in distinguishing nodal nevus cells from ambiguous melanoma micrometastases in sentinel lymph node biopsies [12, 13]. Moreover, the distinct 5-hmC staining profiles of benign and malignant melanocytic proliferations have also been described in tissues of non-melanocytic lineage [14]. Accordingly, the potential for 5-hmC immunoreactivity to distinguish between benign and malignant cellular proliferations makes it a useful marker to explore the pathobiologic significance of small/nevoid cell changes in cutaneous melanoma.

Herein, we describe a unique cohort of primary cutaneous melanomas with small/nevoid (MSCN) subpopulations and explore the immunostaining patterns of 5-hmC within these lesions in comparison to melanomas arising in association with pre-existing nevi (MPEN).

## RESULTS

### Histopathologic and clinical outcome data

Clinical and histopathologic characteristics of each category of melanoma case, including age, gender, anatomic location, average depth, and mitotic rate are summarized in Table 1. There was no significant difference in rate of metastasis between the two categories of melanoma lesions studied, with an overall mean follow-up time of 23.6 months for MPEN cases and 77.2 months for MSCN cases.

**Table 1:** Clinical and histopathological data of primary melanomas with small cell/nevoid subpopulation (MSCN) and those arising in association with pre-existing nevus (MPEN)

	MSCN	MPEN
Total # cases	24	28
Average age (Range)	58.4 (36 to 99)	52.8 (15 to 90)
Gender	Male: 51.4% (13/24)	Male: 46.4% (13 of 28)
	Female: 45.8% (11/24)	Female: 53.6% (15 of 28)
Location of primary	H&N: 4.2% (1/24)	H&N: 7.1% (2 of 28)
	Upper extremity: 12.5% (3/24)	Upper extremity: 17.9% (5 of 28)
	Trunk: 45.8% (11/24)	Trunk: 53.6% (15 of 28)
	Lower extremity: 37.5% (9/24)	Lower extremity: 21.4% (6 of 28)
Average depth of melanoma in mm (Range)	0.89 (0.48 to 1.42)	0.97 (0.3 to 3)
Average mitotic rate per mm-sq (Range)	1.82 (0 to 15)	0.54 (0 to 2)
% Cases w/ nodal metastasis	20% (1 of 5 performed)	25% (3 of 12 performed)*
% Cases w/ distant or locoregional metastases	None	7.1% (2 of 28)
Average follow-up time in months (Range)	77.2 (20 to 99)	23.6 (8 to 37)

* Nodal nevus in 41.7% (5 of 12 SLN biopsies performed)

Review of conventional histology of melanomas with pre-existing nevi (MPEN; *n* = 28) revealed two distinct zones. The first was occupied by a uniform population of large, malignant epithelioid cells containing irregular and angulated nuclei with prominent nucleoli and coarsely clumped, vesicular chromatin patterns; the second was occupied by smaller cells containing round to ovoid nuclei with inconspicuous nucleoli, and evenly distributed, more delicate chromatin (nevus cells). In the majority of cases, the nevic component was deep to the melanoma, while in a minority of cases it was peripheral to or flanked the more centrally localized melanoma. Importantly, gradual transitions between overt melanoma and more nevic components were not encountered, and when present, infiltrating lymphocytes preferentially involved the zones of melanoma but not regions occupied by nevus cells. Melanomas with small/nevoid subpopulations (MSCN, *n* = 24), on the other hand, displayed a range of cytology consisting of a gradual continuum with depth of invasion from more superficial melanoma cells (as described above) to smaller, more nevoid forms. While cells at the base of such lesions were considerably smaller than classic melanoma cells with greater nuclear to cytoplasmic ratio, unlike nevus cells they contained nuclei with irregular and angulated profiles, with not infrequently visible nucleoli and scattered mitoses. Other histologic and clinical parameters are summarized in Table 1.

### 5-hmC immunoreactivity in MPEN *versus* MSCN

**Figure 1:**
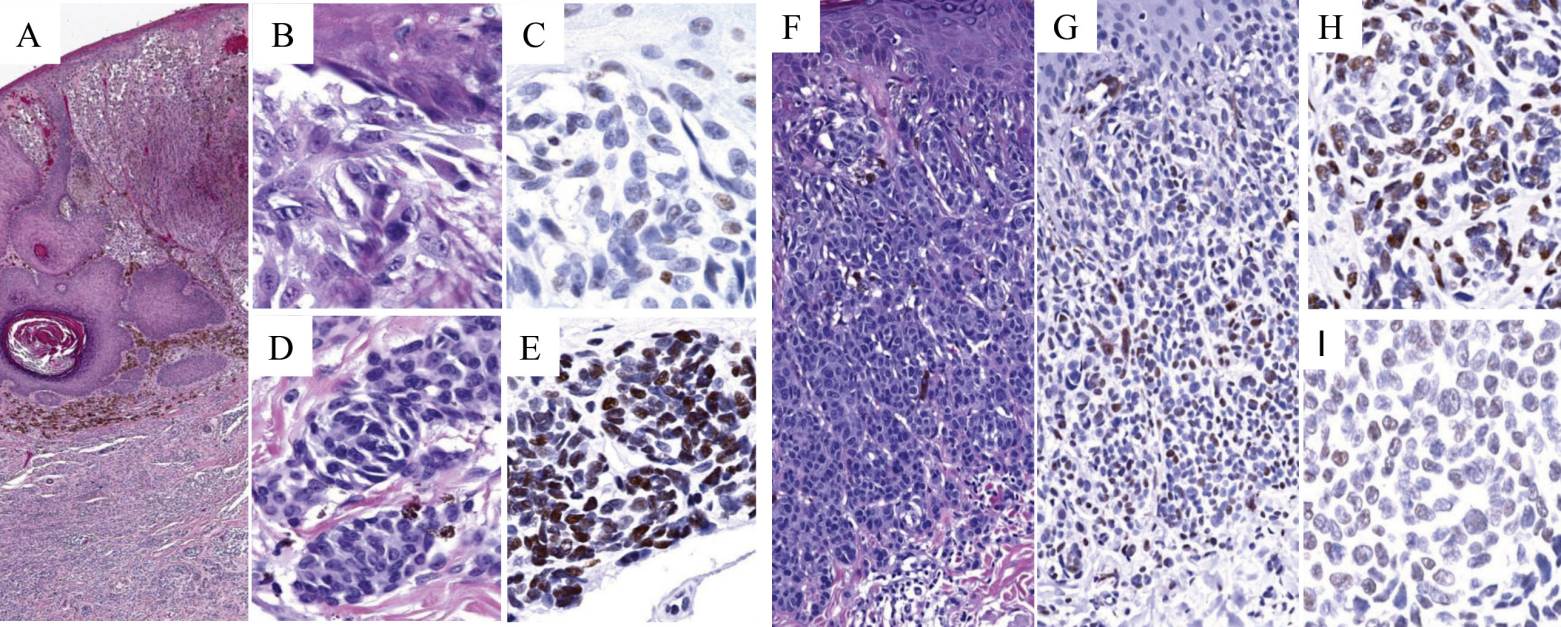
5-hmC immunoreactivity in melanoma arising in association with pre-existing nevus (MPEN; A-E) versus small/nevoid melanoma differentiation (MSCN; F-I) The superficial melanoma component of MPEN (**A.** H&E, 10X; **B.** H&E, 100X) shows complete loss of 5-hmC immunoreactivity (**C.** 5-hmC IHC, 100X), in contrast to associated nevic cells (**D.** H&E, 100X) that demonstrate strong, homogeneous nuclear staining (**E.** 5-hmC IHC, 100X). In contrast, MSCN (**F.** H&E, 20X; **G.** 5-hmC IHC, 20X) shows either intermediate (**H.** 5-hmC IHC, 100X) or near total loss (**I.** 5-hmC IHC, 100X) of 5-hmC immunoreactivity in the small cell component (compare panels H & I with E).

**Figure 2:**
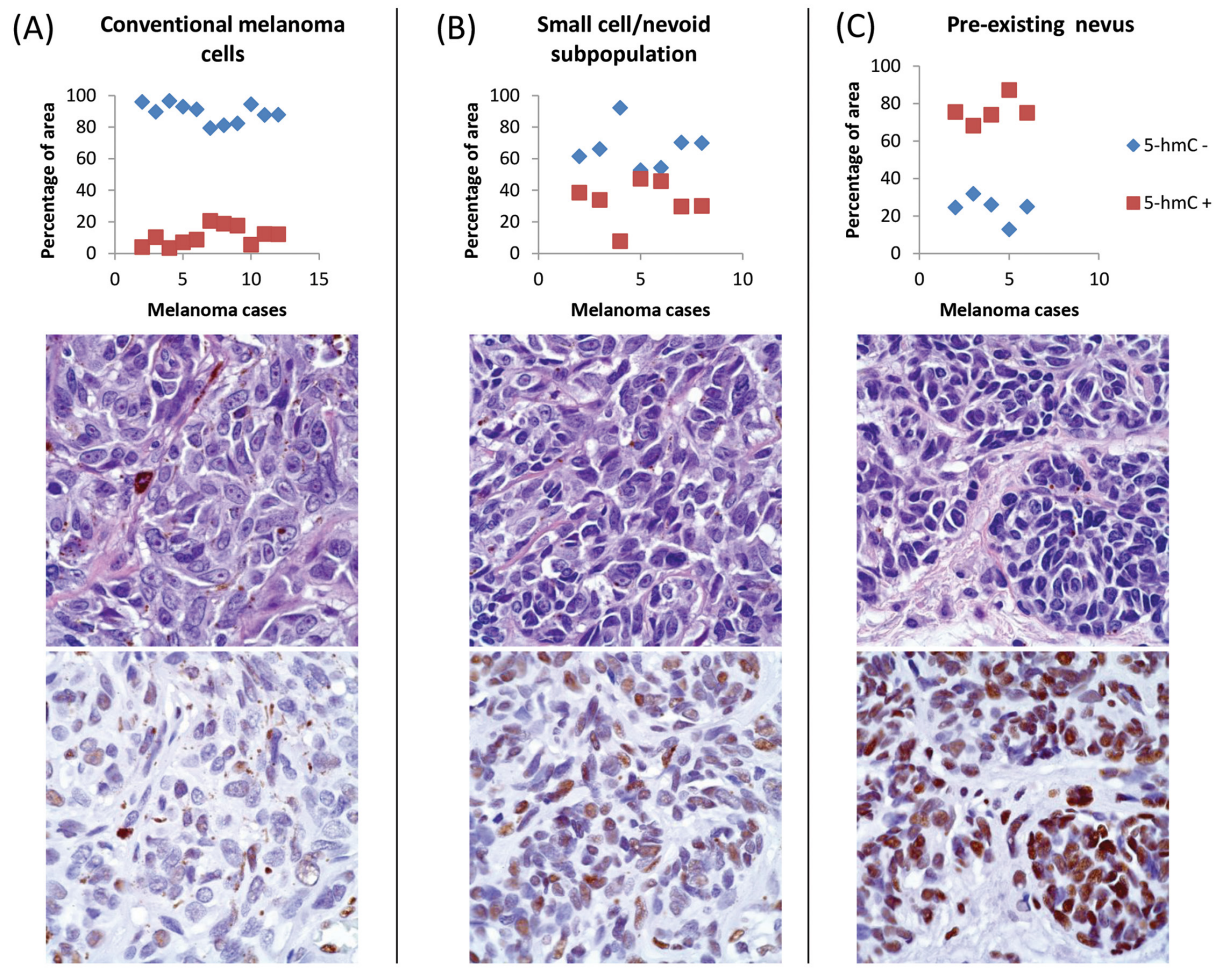
Automated image analysis of distribution of 5-hmC immunoreactivity by ImageJ/IHC Profiler Randomly-selected, representative cases and foci of melanoma (Panel A, *n* = 11), MSCN (Panel B, *n* = 7), and MPEN (Panel C, n=5) revealed quantifiably distinct patterns of 5-hmC positivity and negativity, as plotted as percent area occupied by labeled (red squares) and unlabeled (blue diamonds) cells. Representative H&E and immunohistochemical images below each plot display qualitative immunohistochemical staining characteristics for each analyzed cell subpopulations.

**Figure 3:**
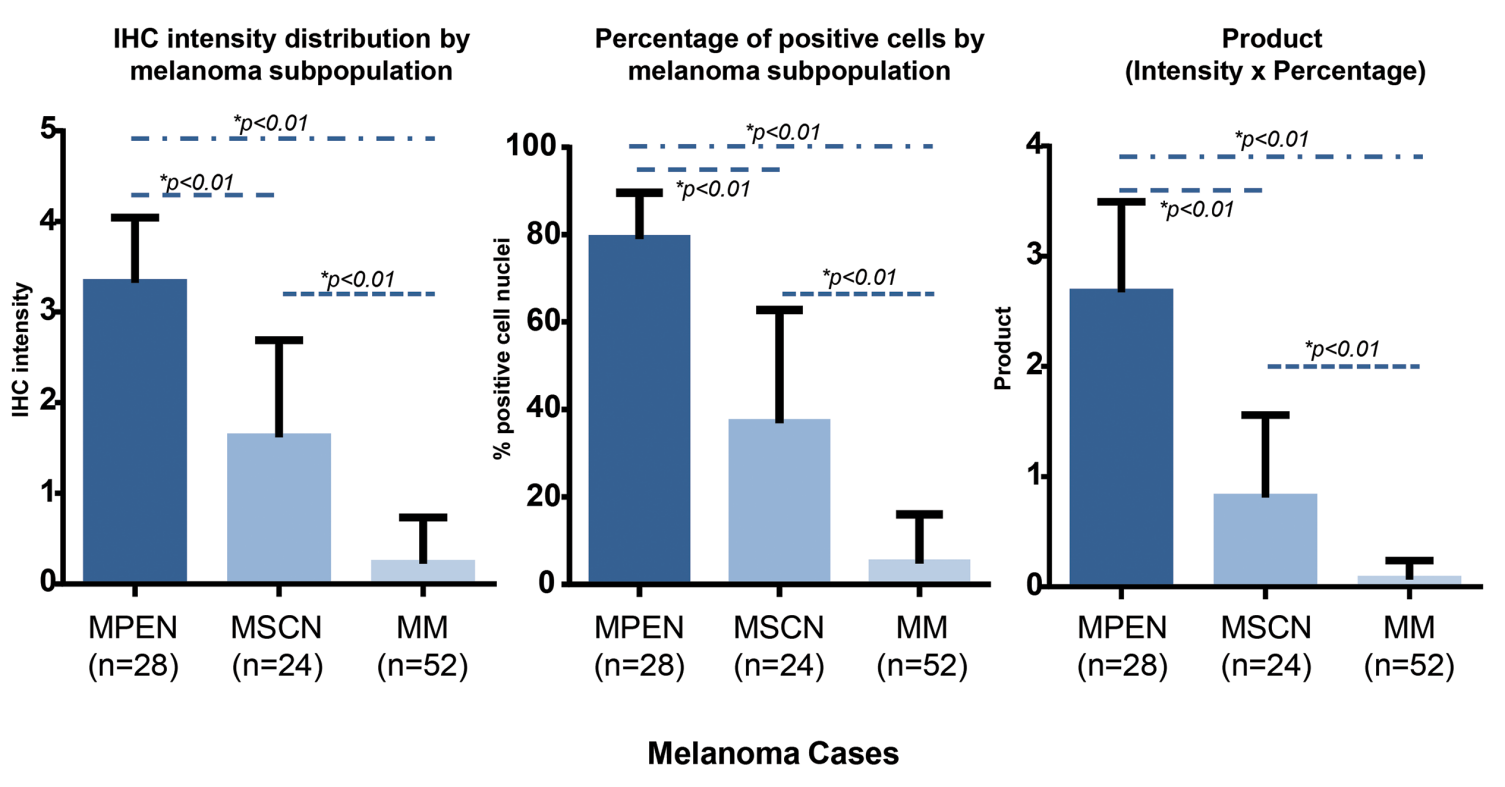
Combined quantitative analyses of intensity and extent of 5-hmC immunoreactivity By manual, semi-quantitative analysis, pre-existing nevi were seen to retain the strongest nuclear intensity for 5-hmC, whereas conventional melanoma cells demonstrated the most significant loss (bar graph to left). Small cell/nevoid subpopulations demonstrated an intermediate 5-hmC staining intensity. Similar trends were observed when the percentage of immunopositive cells were compared (bar graph in middle). The product of the intensity of cell staining and the number of immunopositive cells (bar graph to right) further emphasized the separation possible of MPEN, MSCN, and MM based on 5-hmC immunoreactivity (all p values for all differences <0.01).

Regions of conventional melanoma in all MPEN and MSCN cases (*n* = 52) demonstrated diffuse loss of 5-hmC (mean IHC intensity 0.255; mean percentage of positive cells 5.5%, Figure 1A-1C and Figure 2A). In contrast, pre-existing nevic components associated with melanoma (*n* = 28) showed strong 5-hmC immunopositivity with mean IHC intensity score of 3.32 and mean percentage of positive cells of 79% (Figure 1A, 1D, 1E and Figure 2C). The distinct 5-hmC staining intensities of pre-existing nevic foci *versus* conventional melanoma components was confirmed by automated, digital IHC analysis by ImageJ-IHC Profiler (Figure 2A and 2C). Regions of conventional melanoma transitioning into small cell/nevoid subpopulations in MSCN cases (*n* = 24), on the other hand, demonstrated a 5-hmC staining pattern and intensity that gradually increased with continued invasion and qualitatively corresponded to A reduction in cell size; 5-hmC immunoreactivity remained lost in the overlying, conventional melanoma component (Figure 1F-1I and Figure 2B). Of note, this gradual increase in 5-hmC immunopositivity between zones of conventional melanoma to the small cell/nevoid subpopulation is not seen in conventional melanomas without small cell components (data not shown) [3, 4, 8, 13]. The IHC profile within the small cell/nevoid subpopulation was quantifiably intermediate to that found in conventional melanoma (5-hmC loss) and nevus cells (5-hmC retention), which, too, was confirmed through digital, automated IHC analysis (Figure 2B). Specifically, the mean IHC intensity score for regions of small cell or nevoid subpopulations within the 24 MSCN cases was 1.62 with the mean percentage of positively-stained cells at 37% (Figure 2B, Figure 3). All differences in 5-hmC staining (intensity, percentage of positive cells, and their product) among melanoma, small cell/nevoid, and nevic components were statistically significant (*p* < 0.001) (Figure 3). These findings were validated by semi-automated digital imaging analysis, which further confirmed that the melanoma, small cell/nevoid, and pre-existing nevus components showed distinct immunostaining profiles for 5-hmC (loss, intermediate, and strong retention, respectively). Importantly, there were no cases wherein the 5-hmC immunostaining pattern suggested reclassification of a case from the MPEN to MSCN category or vice versa.

**Figure 4:**
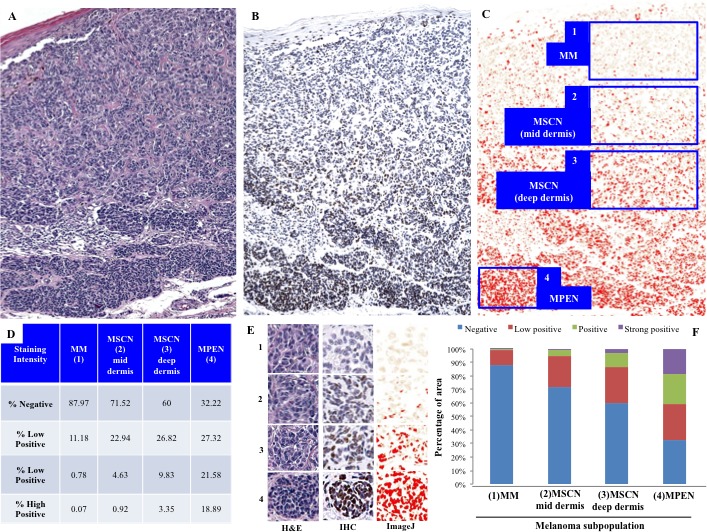
Demonstration of discriminatory ability of 5-hmC staining in a single case (excluded from primary MSCN/MPEN cohorts) containing conventional melanoma, zones of small cell/nevoid differentiation, and pre-existing nevus cells The top panels **A.**-**C.** show a low power view of the melanoma stained by H&E (A, red oval indicates focus of pre-existing nevus), 5-hmC **B.**, and via computer image analysis **C.** Automated quantification by ‘IHC Profiler’ interprets four distinct staining strengths (C: 1, absent in regions of conventional melanoma (MM); 2&3, low positive to positive in regions of MSCN; and 4, high positive in the zone of pre-existing nevus cells (PEN) at the base). Panel **E.** highlights high power views of each of the aforementioned representative zone of cells as seen on H&E, 5-hmC IHC, as well as via computer image analysis. The table **D.** and bar graph **F.** below illustrate the automated quantification of percentage of cells that fell into each intensity category, suggesting that intensity as well as cellular distribution of 5-hmC are enhanced with progression from conventional melanoma (near absence of 5-hmC) to progressively deeper zones of small/nevoid cell differentiation regaining the epigenetic mark.

These findings are best summarized by the routine histologic and 5-hmC immunohistochemical views of a unique case of a melanoma, which was not included in either MPEN or MSCN category (Figure 4). This particular case was composed of conventional melanoma cells that, with depth, gradually transitioned towards a small cell/nevoid subpopulation at the deepest, invasive front and was also arising in association with a separate but adjacent, benign-appearing nevic focus (Figure 4). The 5-hmC immunohistochemical staining profile of each subpopulation (conventional melanoma, small cell nevoid, pre-existing nevus) within this composite lesion, which is further substantiated by the digital, automated interpretation, corresponded to the findings seen in the individual MSCN and MPEN cases (Figure 4).

Of note, two distinct patterns of IHC reactivity were documented in regions of small/nevoid cell changes: approximately 71% (17 of 24) of MSCN cases showed a mean IHC intensity score and percentage of positive cells to be 2.07 and 47.3%, respectively (Figure 1H), while 29% (7 of 24) of MSCN cases had a marked decrease in 5-hmC immunostaining (Figure 1I), with mean IHC intensity score of 0.5, and mean percentage of positive cells 4.2%, in a manner comparable to the overlying melanoma. Although the basis for this apparent dichotomy in 5-hmC immunoreactivity in areas of small/nevoid subpopulation is not entirely clear, it was noted that MSCN cases with intermediate staining tended to be more superficial and less mitotically active than the MSCN cases with markedly decreased staining. These differences, however did not reach statistical significance in the limited number of cases under study (Table 2).

**Table 2:** Histopathological features compared between cases of melanoma with small cell/nevoid subpopulations (MSCN) demonstrating intermediate/partial increase in 5-hydoxymethylcytosine immunopositivity to those showing continued loss of 5-hmC

	MSCN w/ intermediate 5-hmC positivity	MSCN w/ 5-hmC loss
% of MPM cases	70.8 (17 of 24)	29.2% (7 of 24)
Average depth of primary in mm (Range)*	0.85 (0.4 to 1.2)	0.93 (0.5 to 1.19)
Average mitotic rate per mm-sq (Range)*	0.82 (0 to 7)	3.6 (0 to 15)

* Differences not statistically significant

## DISCUSSION

Our data extend that of previous studies and indicate that ‘loss’ of 5-hydroxymethylcytosine (5-hmC) may be a useful biomarker for distinguishing melanoma with small cell/nevoid components from pre-existing nevus cells. While prior studies demonstrate the use of 5-hmC to distinguish such components in separate cases of benign *versus* malignant melanocytic lesions [3, 8, 12, 13], the present study illustrates its potential diagnostic utility in discriminating between pathobiologically distinct melanocytic subpopulations within a single lesion. Prognostically, because persistence of pre-existing nevus cells very closely resembles nevoid or small cell subpopulations, 5-hmC immunoreactivity may be a useful diagnostic adjunct in clarifying benign from malignant cells for the purposes of microstaging, particularly when either is present at the deepest, invasive front of the tumor.

Our MSCN cases revealed two unique patterns of 5-hmC immunostaining within the small/nevoid cell populations. Slightly less than one third of cases demonstrated marked loss of 5-hmC akin to that of the larger, overt melanoma cells. In contrast, more than two-thirds of MSCN cases demonstrated an intermediate (weak, or partial loss of) 5-hmC immunopositivity within the small cell/nevoid subpopulations that, by intensity of staining, was distinctly stronger than that of the contiguous, overlying melanoma cells (complete loss) yet remarkably fainter than that of benign associated nevic cells (strong positivity) of the MPEN cases. Digital, quantitative immunohistochemical analysis provided computer-assisted validation of the distinct 5-hmC immunohistochemical staining profiles (‘loss’ in melanoma; ‘intermediate’ in small cell/nevoid cells; ‘retained’ in pre-existing nevus) while also demonstrating the gradient of decreasing 5-hmC positivity characteristic of the small cell/nevoid component with increasing dermal depth.

Previous tissue microarray analyses have shown that those melanomas with the most profound loss of 5-hmC by immunohistochemistry corresponded to those patients who suffered the worst melanoma-related outcomes [8]. In addition, prior studies have demonstrated significant differences in the methylation and hydroxymethylation status of genes involved in pathways known to play a role in melanomagenesis and other cancer pathways through analysis of immunoprecipitated hydroxymethylated DNA coupled with deep sequencing comparing nevi and melanoma [8]. We have also previously shown that restoration of the ‘5-hmC landscape’ *via* overexpression of TET2 in xenograft models resulted in more indolent, less invasive melanomas, suggesting an important, epigenetically-mediated tumor-suppressive role for TET2 activity and 5-hmC [8]. Taken in context, our observations that MSCN subpopulations may retain some capacity to express 5-hmC raise the possibility that small/nevoid cells may reflect a more indolent, less virulent subset of melanoma cells [7]. This hypothesis is in keeping with that raised by the gradual reduction of HMB-45 and Ki-67 reactivity within so-called ‘paradoxically maturing’ melanoma cells as observed and reported by Ruhoy et al. [7].

After careful review of our cases, we found that the presence of intermediate 5-hmC levels within the small cell/nevoid subpopulation at the base of the invasive nodule could assist in differentiating this nevoid subpopulation from pre-existing nevus. Importantly, this observation also raises the clinically and prognostically-relevant question of whether a Breslow depth measured to the nevoid/small cell 5-hmC-intermediate subpopulation, as opposed to the deepest, conventional, 5-hmC negative melanoma cells, reflects the true prognosis of this cytologically heterogeneous lesion [7]. For these reasons, further studies of larger cohorts of clinically annotated MSCN cases are now indicated to assess the prognostic accuracy and significance of microstaging such cases to the full depth of a 5-hmC-positive small cell/nevoid subpopulations (as opposed to only the conventional 5-hmC negative melanoma population). The clinical and economic significance of identifying and remediating the potential systematic over-treatment of cases wherein the depth may be overestimated and, conversely, the under-treatment of cases whose prognosis may be underestimated is clear. Until such data become available, however, microstaging MSCN cases to the deepest melanocytic cell, despite some degree of 5-hmC positivity, is appropriate and would allow for greater sensitivity in identifying the potentially most dangerous lesions.

Although not statistically significant in this limited cohort, it is worth noting that none of our MSCN cases developed locoregional or metastatic disease in contrast to two of our MPEN cases. The limited amount of follow-up time present, particularly among the MPEN cases, may further preclude our ability to detect a significant difference in outcome between MSCN and MPEN cases. Nonetheless, it is worth reiterating that the prognosis of MPEN lesions to date does not appear to differ from that of *de novo* melanomas [3]. Given the limited number of MSCN cases in our cohort, our findings should be interpreted with caution. Nonetheless, the hypothesis that cutaneous melanomas containing small cell/nevoid subpopulations at the invasive front, particularly when they demonstrate 5-hmC intermediate immunopositivity, may have a better prognosis than conventional or nevus-associated melanomas deserves further investigation. Molecular characterization at the cellular level, comparing conventional and small cell/nevoid subpopulations within a single MSCN case may provide additional, clinically relevant insight.

‘Loss of 5-hmC’ is thought to reflect dysfunction of the TET family of 5-methylcytosine (5-mC) hydroxylases, which perform the critical oxidation steps that convert 5-mC to 5-hmC and other derivatives along the recently described pathway of active DNA de-methylation [10]. This critical epigenetic function is hypothesized to provide a potential mechanism that would enable the removal of the methyl group from ‘incorrectly’ methylated sites, earning TET the epithet, ‘guardian’ of DNA methylation fidelity [11]. In addition, observations to date strongly suggest that the loss of 5-hmC is highly specific to malignant as well as normal, regenerative, stem-like cells in most, if not all, human organ systems [14]. 5-hmC immunostaining of normal epithelial tissues such as the skin, for instance, demonstrates that regenerative, basal keratinocytes are negative; yet as they mature and rise through the epidermal strata, keratinocyte nuclei progressively acquire 5-hmC with increasing differentiation, with the strongest nuclear immunoreactivity in the uppermost strata [14]. For these reasons, it is also plausible that increasing 5-hmC content may serve as an epigenetic correlate of increasing biological differentiation. Accordingly, its ‘loss’ in the context of malignancy may reflect a biologically dedifferentiated state and/or increased replicative capacity. Such data, by analogy, provide evidence to support our hypothesis that intermediate patterns of 5-hmC immunoreactivity in the context of small cell or nevoid subpopulations may reflect a more differentiated and, in part by convention, a potentially less virulent ‘melanoma’ state. Indeed, ‘well-differentiated’ variants of most carcinomas are known to confer a much better prognosis than their ‘poorly-differentiated’ counterparts. If this hypothesis is true with respect to melanoma and small cell/nevoid subpopulations, harnessing the epigenetic machinery responsible for biological differentiation and therapeutically repurposing it to induce indolence may be worthy of investigation.

Recent studies have attempted to characterize the prevalence of somatic mutations to *TET2*, a key member of the TET family, in cutaneous melanomas [15]. However, this fraction is significantly less than the prevalence of dysfunctional TET2, as is indicated by the loss of 5-hmC in most, if not all, conventional primary cutaneous melanomas. Moreover, the subtle but quantifiable increase in 5-hmC immunoreactivity within the small cell components of our series of cutaneous melanomas all suggest that mechanisms beyond mutations in the *TET* gene itself are likely responsible for its dysfunction in this context, as suggested by previous studies [8]. In fact, recent experimental data support that abnormal metabolic pathways and consequent oncometabolite accumulation can, alone, inhibit proper function of this critical epigenetic regulator [17, 18]. Thus, it remains possible that epigenetic mechanisms and/or microenvironmental influences may be, in part, responsible for partial 5-hmC levels within the small cell variants of melanoma cells.

It is known that the cutaneous microenvironment differs at varying depths based on the presence or absence of certain cell-cell or cell-matrix interactions, cytokines, or growth factors [19]. In addition, specific differences in the tumor microenvironment, such as localized hypoxia, have also been proposed to modify gene expression profiles that favor enhanced invasiveness and virulent behavior [20]. Thus, it is tempting to speculate whether changes in the cutaneous microenvironment may also play, in part *via* epigenetic mechanisms, an influential role in the biology and small/nevoid cells observed in a subset of melanomas, as has been previously suggested [7). It is plausible that melanocytes harness a uniquely flexible physiologic epigenetic program, given their duty to modify gene expression in a timely fashion in response to environmental exposures and cues such as ultraviolet radiation. However, the role of epigenetic mechanisms in normal melanocyte physiology has yet to be established.

In summary, our study of immunohistochemical staining patterns of the functionally and prognostically-relevant epigenetic mark, 5-hmC, in this unique collection of primary cutaneous melanomas highlights epigenomic heterogeneity within a subset of vertical growth phase melanomas that corresponds to and distinguishes between *bona fide* melanoma, pre-existing nevus, and small cell or nevoid mimicry within a single composite lesion. In addition to the biological implications of our findings, there now exists potential utility of immunohistochemical staining with 5-hmC to enhance diagnostic accuracy and precision in selected cases where small/nevoid melanoma cells *versus* presence of nevus cells provokes diagnostic uncertainty. Importantly, further application and exploration of the clinical and prognostic utility of this assay in the assessment of melanomas demonstrating so-called polyclonism or of other diagnostically challenging melanocytic lesions, in the context of primary skin sections or positive sentinel lymph node specimens, are now indicated.

## MATERIALS AND METHODS

### Histopathologic samples & data

This study was approved by the Institutional Review Board of the Brigham and Women's Hospital (Boston, MA) and the Royal Surrey County Hospital (Surrey, UK). A total of 52 cases of primary cutaneous melanomas (*n* = 52) were retrieved from the pathology archives. Twenty-four of these cases were intentionally, prospectively collected by the investigators (MC, MCM) based on the presence of smaller, nevoid cells at the invasive front underlying a conventional primary cutaneous melanoma lesion (*n* = 24, MSCN). Cases of melanomas associated with pre-existing nevus cells were also retrieved from the pathology archives of the two institutions (*n* = 28, MPEN). Detailed histopathologic data of each of the primary melanomas, including synoptic melanoma diagnostic/prognostic information, such as depth, ulceration, and mitotic rate, were obtained for each case based on routine H&E evaluation. The H&E-stained sections, prior diagnoses, and prognostic features were independently reviewed and confirmed by two study dermatopathologists (CGL, GFM).

### Immunohistochemistry & quantitative scoring

Immunohistochemistry (IHC) for 5-hydroxymethylcytosine (5-hmC) was performed on all cases (*n* = 52) as we previously described [8]. Sections were incubated overnight with rabbit-anti-5-hmC (Active Motif, Carlsbad, CA; 1:5,000 dilution), washed, and subsequently incubated with a peroxidase-linked anti-mouse IgG (Vector Laboratories, Burlingame, CA; 1:200 dilution). The sections were then treated with the corresponding hydrogen peroxide substrate kit (Vector Laboratories, Burlingame, CA) and counterstained in hematoxylin and clarifying solution (Fisher Scientific Company, Kalamazoo, MI). Appropriate isotype-matched antibody controls and tissue controls were employed for all experiments.

5-hmC staining was scored based on previously published methodology [9]. In brief, immunoreactivity was quantified based on the nuclear staining intensity (0-4; 0 = absent; 4 = dark brown reactivity involving the entire nuclear profile, and 1 through 3 representing semiquantitative intermediates [1 = faint tan; 2 = light brown; 3 = medium brown]. In addition, the percentage of 5-hmC-positive cells of melanocytic lineage, as assessed over representative 1-mm^2^ fields, was also determined. Fields were selected based on the presence of key histologic features relevant to the study (i.e. small/nevoid melanoma cells, pre-existing nevus, melanoma compartment), as assessed and determined by H&E examination alone. A total of five randomly selected but representative high power fields were examined in each histologic section. When heterogeneity in staining intensity within the melanocytic cells was encountered, the most predominant staining intensity pattern was numerically represented, but great care was taken to capture and represent each possible profile in the multiple fields studied. All semiquantitative immunoreactivity scoring was performed by one investigator (JJL) and a random subset of MSCN (*n* = 10) and MPEN (*n* = 12) cases was reviewed by a second investigator (CGL) to ensure concordance. Reviewers were blinded to the diagnosis of each case prior to their evaluation.

Further validation of the manual, blinded, semiquantitative immunohistochemical scoring was also performed on a randomly selected subset of cases from each category (7 MSCN cases, 5 MPEN cases) using the automated digital image analysis software ImageJ (Image J, U. S. National Institutes of Health, Bethesda, Maryland, USA) and the IHC Profiler plug-in [15, 16].

### Quantitative analysis

Clinical and histopathologic data between MSCN and MPEN cases were compared using two-sample *t*-tests using StatPlus:Mac version 5.8.2.0 (AnalySoft, London, United Kingdon). Immunohistochemical staining scores were compared between specific foci of interest (i.e. melanoma, pre-existing nevus, melanomas with small/nevoid subpopulation, etc.) by performing one-way ANOVA analysis using GraphPad Prism version 6 (GraphPad Software, La Jolla, CA).

### Abbreviations

5-hydroxymethylcytosine (5-hmC); H&E (hematoxylin & eosin); MPEN (melanoma arising in association with pre-existing nevus); MSCN (melanoma with small cell or nevoid subpopulation); TET (Ten-Eleven Translocase)
